# CXCL13 Damages Blood Spinal Cord Barrier by Promoting RNF6/Sqstm1‐Ubiquitination Induced Autophagy in Experimental Allergic Encephalomyelitis

**DOI:** 10.1002/advs.202414550

**Published:** 2025-04-15

**Authors:** Jingjing Han, Rui Hong, Cong Cao, Wanhua Feng, Wei Zhuang, Gui Wang, Jingchao Tang, Ya Yang, Chu Zhang, Aihua Zhou, Xuebin Qu

**Affiliations:** ^1^ Department of Basic Medical Science Jiangsu Medical College Yancheng Jiangsu 224005 China; ^2^ The Fourth People's Hospital of Yancheng Yancheng Jiangsu 224003 China; ^3^ Department of Cell Biology and Neurobiology Xuzhou Medical University Xuzhou Jiangsu 221004 China; ^4^ Emergency Medicine Department of the Affiliated Hospital of Xuzhou Medical University Xuzhou Jiangsu 221000 China; ^5^ Group Health Section The Affiliated Yancheng Maternity & Child Health Hospital of Yangzhou University Yancheng Jiangsu 224000 China

**Keywords:** autophagy, blood spinal cord barriers, CXCL13, experimental allergic encephalomyelitis, multiple sclerosis, RNF6

## Abstract

The damage of blood spinal cord barrier (BSCB) is contributing to the disruption of immune microenvironment within central nervous system during the progression of multiple sclerosis (MS) and experimental allergic encephalomyelitis (EAE). Nevertheless, the underlying mechanisms responsible for barrier impairment remain inadequately understood. Here, by analyzing the protein profiles in peripheral blood serum, chemokine (C‐X‐C motif) ligand 13 (CXCL13) was identified to be increased with the progression of MS and EAE. The absence of CXCL13 resulted in alleviation of EAE symptoms, as evidenced by a reduced clinical score, decreased barrier damage, as well as diminished demyelination and inflammatory response in the spinal cord. In the BSCB model, CXCL13 was found to impair barrier structure and function in a dose‐ and time‐dependent manner, which was associated with exacerbated autophagy in endothelial cells, while the application of autophagy inhibitors partially mitigated this damage. Mechanistically, CXCL13 enhanced the expression of RNF6, an E3 ubiquitin‐protein ligase, facilitating the conjugation to Sqstm1 for the ubiquitination at the K314 residue. These findings suggest that CXCL13 significantly contributes to the impairment of the BSCB by promoting RNF6/Sqstm1‐ubiquitination‐induced autophagy during the progression of EAE, thereby offering a promising diagnostic and therapeutic target for MS.

## Graphical abstract

In multiple sclerosis, the disruption of the blood‐spinal cord barrier (BSCB) induced by CXCL13 facilitates the infiltration of inflammatory cells into the central nervous system, resulting in demyelination and neuronal injury. Mechanistically, the deleterious impact of CXCL13 on the BSCB is associated with a reduction in tight junction protein expression and the promotion of endothelial cell autophagy by up‐regulating RNF6 to ubiquitinate Sqstm1.

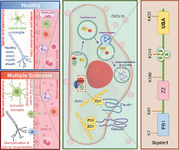

## 1 Introduction

Multiple sclerosis (MS), as a chronic inflammatory autoimmune disease that occurs in the central nervous system (CNS), affects millions of people worldwide.^[^ ^1^ ^]^ MS is characterized by pathological features including demyelinating plaques in the white and gray matter of the brain and spinal cord, caused by damage to myelin sheaths and oligodendrocytes.^[^ ^2^ ^]^ Until now, the clear etiology and pathogenesis of MS are still not fully understood. Current researches suggest that the occurrence and development of MS are the result of a combination of multiple factors, including autoimmunity, viral infection, genetic predisposition, environmental conditions, and individual susceptibility, among which autoimmune inflammatory response is the main trigger, as destabilizing the immune microenvironment balance, promoting diffuse infiltration of inflammatory cells, activating microglia and astrocytes, and attacking the CNS myelin sheath for a result of myelin sheath loss, axonal injury, and atrophy of white and gray matter.^[^ ^3^ ^]^

During the occurrence and development of MS, the damage to the blood‐spinal cord barrier (BSCB) is closely related to the disruption of the CNS immune microenvironment and the infiltration of inflammatory cells.^[^ ^4^ ^]^ The barrier damage is not only one of the early symptoms of MS, but also participates in the later stages of disease progression such as demyelination, axonal injury, and neuronal degeneration.^[^ ^5^ ^]^ These selective barriers between the blood system and the central nervous system are composed of continuous microvascular endothelial cells and tight junctions between cells, intact basal membrane, pericytes, and the terminal glial membrane formed by astrocytes. An intact barrier can strictly control the entry and exit of substances into or out of the brain parenchyma, maintaining the stability of the CNS environment, while barrier damage leads to a large number of inflammatory cells infiltrating the CNS, inducing pathological activation of astrocytes and microglia to secrete excessive inflammatory factors such as IL‐1, IL‐6, TNF, etc., further damaging the structure and function of barriers and exacerbating the deterioration of the lesion.^[^ ^6^ ^]^ During the pathogenesis of MS and experimental autoimmune encephalomyelitis (EAE), the expression of tight junction proteins is reduced, and the degree of damage to tight junctions is closely related to the disease progression.^[^ ^7^ ^]^ Enhancing the expression of tight junction molecules on brain endothelial cells can inhibit the development of CNS autoimmune diseases.^[^ ^8^ ^]^ However, the causes and mechanisms of structural damage and functional impairment of the barrier in MS and EAE are still unclear.

Sheikh et al. found that the serum of MS patients could impair the energy metabolism of endothelial cells in the barrier model in vitro, leading to dysfunction of tight junction proteins and increased barrier permeability. The underlying mechanism of this result was related to the increased release of reactive oxygen species, down‐regulation of anaerobic metabolism pathways, and mitochondrial damage.^[^ ^9^ ^]^ Research showed that cytokines in serum played an important role in BSCB structural damage and dysfunction. In order to explore the key factors that damage the BSCB during the progress of MS, this study screened and identified the chemokine (C‐X‐C motif) ligand 13 (CXCL13) was at an abnormally high level in the peripheral blood serum from both clinical MS patients and EAE mice. CXCL13, a member of the CXC chemokine family, plays an important role in lymphocyte development and homing and is also associated with the occurrence and maintenance of tissue inflammation. Current research has shown that an unbalanced level of CXCL13 is involved in the occurrence of autoimmune diseases such as rheumatoid arthritis, MS, and systemic lupus erythematosus.^[^ ^10^ ^]^ In the peripheral blood, cerebrospinal fluid, and CNS demyelinating plaques of relapsing MS patients, the levels of CXCL13 were significantly increased, while CXCL13 was reduced during the remitting period, accompanied by a decrease of CNS inflammatory cells.^[^ ^11^ ^]^ Although CXCL13 has been considered as a biomarker for the occurrence and development of MS,^[^ ^11^, ^12^ ^]^ its role and mechanism in BSCB injury are not yet fully understood.

Currently, the clinical use of immunosuppressants and immunomodulatory drugs can mitigate the onset and progression of MS;^[^ ^13^ ^]^ however, preventing and reversing the disease's progression remains challenging.^[^ ^14^ ^]^ Thus, this study explores the role and mechanism of inflammatory factor CXCL13 in inducing BSCB structural and functional damage during EAE, in order to provide empirical evidence to elucidate the pathogenesis of MS and identify potential targets for prevention and treatment.

## 2 Results

### 2.1 BSCB Integrity was Damaged as EAE Progressing

**Figure 1:**
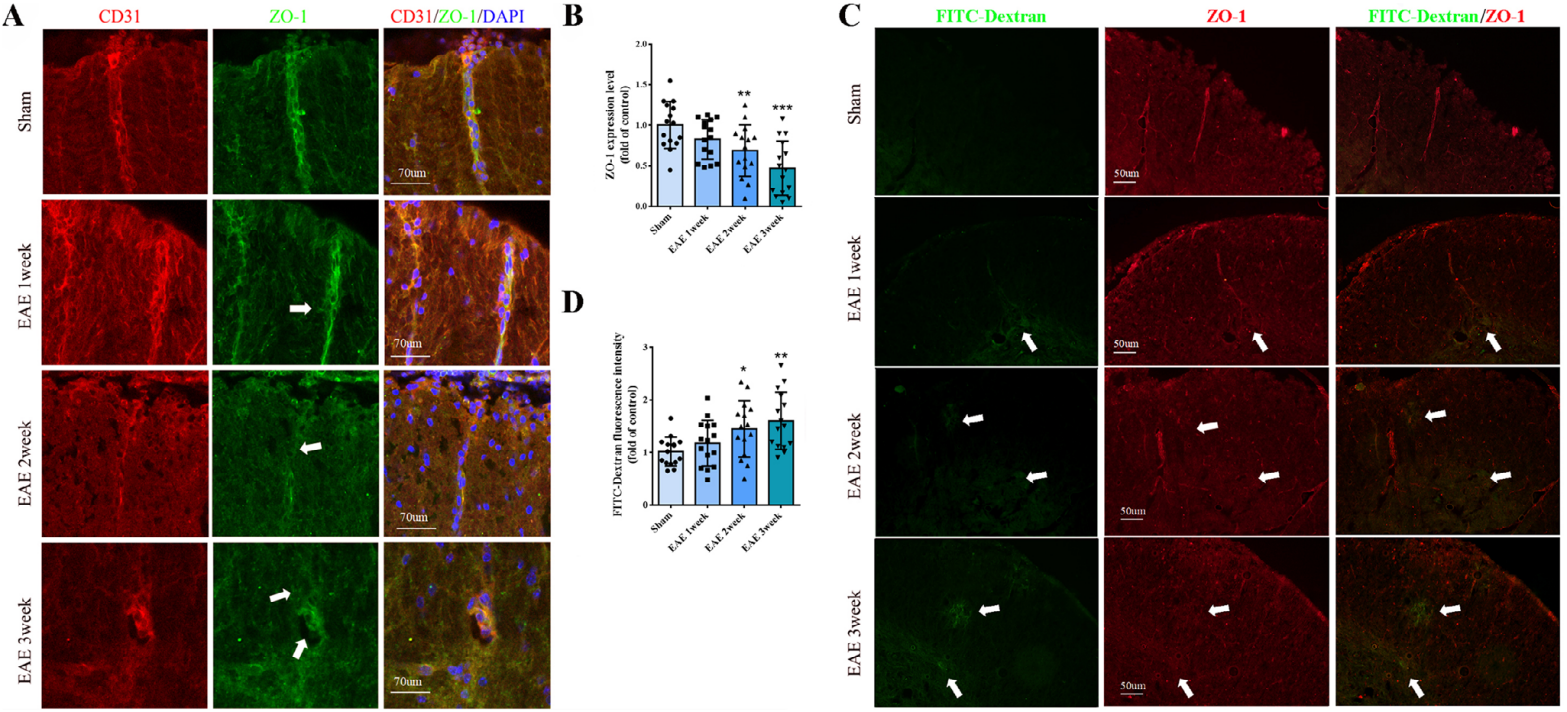
BSCB is damaged in EAE mice. A) Immunofluorescence staining of CD31 and ZO‐1 in spinal cords from EAE mice at 1, 2, and 3 weeks post‐MOG immunization. Bar, 70 µm. Arrows show disappeared ZO‐1. B) Quantification analyses of ZO‐1 in A. C) Observation of fluorescein leakage in spinal cords from EAE mice. Bar, 50 µm. Arrows show disappeared ZO‐1 and leakage sites. D) Quantification analyses of fluorescein leakage in C. *n*  =  15. Data are presented as the mean ± SD. *p <0.05, **p <0.01, ***p < 0.001 versus Sham.

To investigate the disruption of the BSCB during the pathogenesis of EAE, the integrity of the BSCB was assessed in EAE mice at 1, 2, and 3 weeks following MOG immunization. The findings indicated a significant reduction in the levels of zonula occludens‐1 (ZO‐1), a tight junction protein within the BSCB, accompanied by a localized loss in EAE mice compared to the Sham group (**Figure** **1**A,B). Following intravenous administration of FITC‐Dextran, fluorescence was observed to permeate and accumulate around the spinal cord blood vessels in EAE mice (Figure 1C), with increased fluorescein leakage correlating with the progression of EAE (Figure 1D). These results suggest that the structural integrity of the BSCB is compromised during EAE progression.

### 2.2 CXCL13 was Related to the Progress of MS and EAE

**Figure 2:**
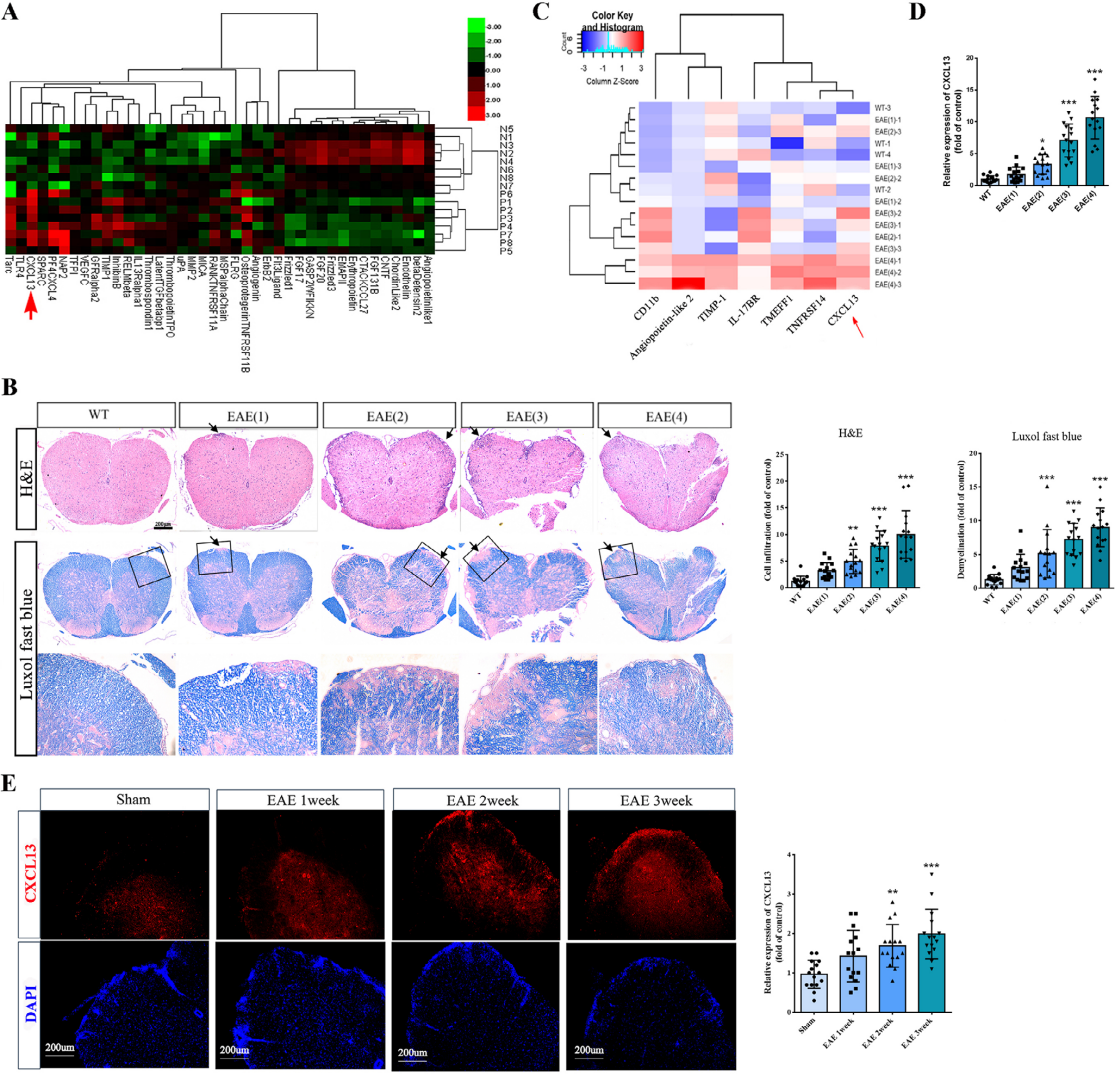
CXCL13 is up‐regulated in MS patients and EAE mice. A) Clustered heat map shows the differential proteins in the peripheral blood serum between MS patients (P1–8) and healthy volunteers (N1–8). Arrow shows CXCL13. B) H&E staining and LFB staining of spinal cord sections from the WT mice and the EAE mice with different identified scores. Quantification analyses are on the right. The numbers in brackets indicate the different EAE scores. Arrows show lymphocyte infiltration and demyelination. The enlarged images are shown below. Scale bar, 200 µm. C) Clustered heat map shows the differential proteins in the peripheral blood serum between the WT mice (*n*  =  4) and the EAE mice with different scores (*n*  =  3 *for* each group). The numbers in brackets indicate the different EAE scores. −1, −2, −3, and −4 indicate replicates. Arrow shows CXCL13. D) Quantification analyses of CXCL13 in the serum. E, Immunofluorescence staining of CXCL13 in spinal cords from EAE mice at 1, 2, and 3 weeks following MOG immunization. Quantification analyses are on the right. Bar, 200 µm. *n*  = 15. Data are presented as the mean ± SD. *p <0.05, **p <0.01, ***p < 0.001 versus WT or Sham.

To explore the specific cytokines involved in BSCB damage, peripheral blood serum samples were collected from eight clinical MS patients for protein mass spectrometry analysis, and the data indicated elevated levels of CXCL13 in MS patients (**Figure** **2**A, Table S1, Supporting Information). In addition, EAE mice induced by MOG_35‐55_ were categorized based on varying clinical scores (ranging from 1 to 4 points), and the extent of inflammatory cell infiltration and demyelinating lesions in the spinal cord of EAE mice increased in correlation with higher EAE scores (Figure 2B). The protein mass spectrometry analysis of peripheral blood serum from EAE mice revealed a significant elevation in CXCL13 levels, which further exhibited a progressive up‐regulation correlating with increasing EAE scores (Figure 2C,D;Table S2, Supporting Information). Similarly, immunofluorescence staining demonstrated a marked increase in CXCL13 levels within the spinal cord of EAE mice compared to the Sham group, with a further incremental rise observed alongside EAE progression (Figure 2E). These findings indicate a potential involvement of CXCL13 in the pathogenesis of MS and EAE.

### 2.3 CXCL13 Deficiency Alleviated EAE and BSCB Damage

**Figure 3:**
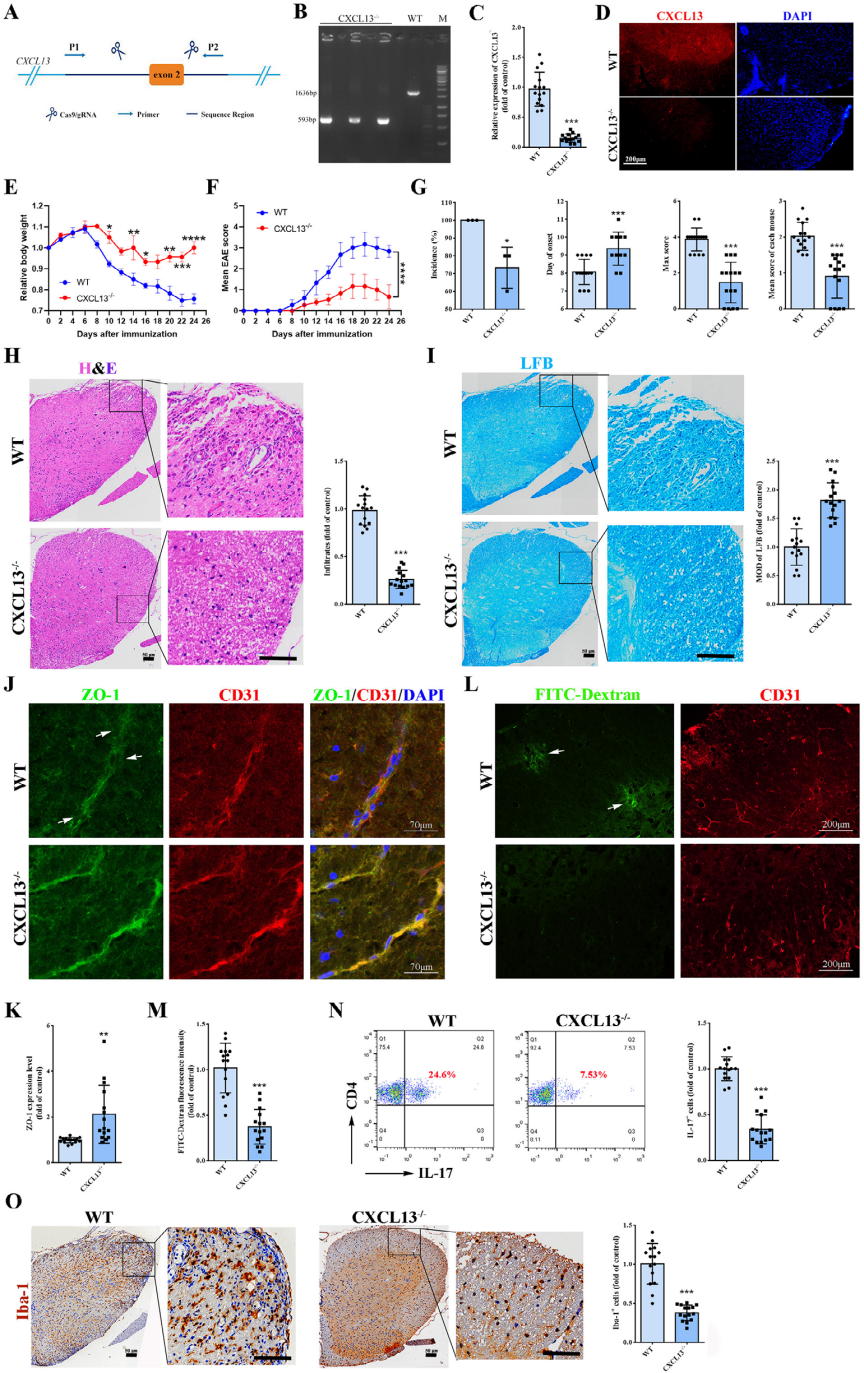
CXCL13 deficiency alleviates BSCB damage in EAE mice. A) The constructing strategy of CXCL13^−/−^ mice. B) The genotyping for CXCL13^−/−^ mice. C) The levels of CXCL13 in plasma. D) Immunofluorescence staining of CXCL13 in spinal cords. Bar, 200 µm. E) The ratio of body weight normalized to initial weight of each mouse. F) Mean scores of EAE mice. G) Quantification analyses of incidence, day of onset, max score, and mean score of EAE mice. H) H&E staining and quantification analyses of spinal cord sections from EAE mice. I) LFB staining and quantification analyses of spinal cord sections from EAE mice. J) Immunofluorescence staining of CD31 and ZO‐1 in spinal cords from EAE mice. Arrows show disappeared ZO‐1. K) Quantification analyses of ZO‐1 in J. L) Test of fluorescein leakage in spinal cords from EAE mice. Arrows show disappeared ZO‐1 and leakage sites. M) Quantification analyses of fluorescein leakage in L. N) Flow cytometric analysis of IL‐17^+^ cells among CD4^+^‐gated cells from spinal cord‐infiltrated lymphocytes in EAE mice 20 days after MOG immunization. O) Immunohistochemical staining and quantification analyses of Iba‐1^+^ cells in spinal cords from EAE mice. *n*  = 15. Data are presented as the mean ± SD. *p <0.05, **p <0.01, ***p < 0.001.

To elucidate the role of CXCL13 in the pathogenesis of EAE, CXCL13‐deficient (CXCL13^−/−^) mice were generated (**Figure** **3**A–D), and subsequently immunized with MOG_35–55_ to induce EAE. In comparison to the WT group, CXCL13^−/−^ EAE mice exhibited reduced body weight loss (Figure 3E) and attenuated EAE symptoms (Figure 3F), characterized by a lower incidence rate, delayed disease onset, and decreased EAE scores (Figure 3G). Histopathological examination of spinal cord sections revealed that CXCL13^−/−^ EAE mice displayed reduced inflammatory cell infiltration (Figure 3H) and decreased demyelination (Figure 3I). In the spinal cords of CXCL13^−/−^ EAE mice, there was an observed up‐regulation of ZO‐1 expression (Figure 3J,K) and a concomitant reduction in fluorescein leakage around the blood vessels (Figure 3L,M). Furthermore, compared to WT EAE mice, the CXCL13^−/−^ EAE mice exhibited significantly lower proportions of IL‐17^+^ lymphocytes in the spinal cord (Figure 3N), which corresponded with a reduced presence of Iba‐1^+^ cells (Figure 3O). Collectively, these findings suggest that the absence of CXCL13 mitigates BSCB damage, inflammation, and the severity of EAE.

### 2.4 CXCL13 Damaged Tight Junction Integrity of BSCB In Vitro

**Figure 4:**
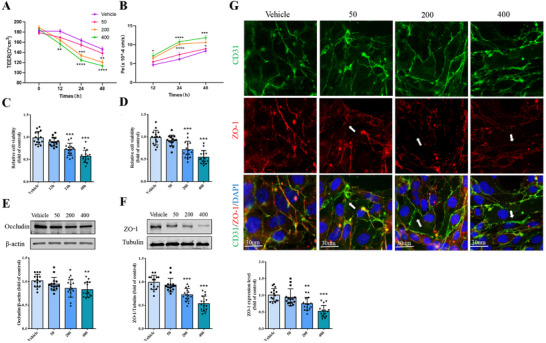
CXCL13 damages BSCB in vitro. Various concentrations of CXCL13 (0, 50, 200, and 400 ng mL^−1^) were introduced into the BSCB culture medium to evaluate their effects at distinct time intervals (12, 24, and 48 h). A) TEER values are recorded for the measure of junctional tightness. B) Fluorescence permeability (Pe) is measured for evaluating BSCB integrity. C,D) Cell viability testing by CCK8 in bEnd.3 cells. E,F) Western blot and quantification analyses of Occludin and ZO‐1 in bEnd.3 cells. G) Immunofluorescence staining of CD31 and ZO‐1 in bEnd.3 cells. Arrows show disappeared ZO‐1. Quantification analyses are shown below. *n*  = 15. Data are presented as the mean ± SD. *p <0.05, **p <0.01, ***p < 0.001, ****p < 0.0001 versus Vehicle.

To examine the deleterious impact of CXCL13 on the BSCB, an in vitro model was established by coculturing the microvascular endothelial cell line bEnd.3 with primary astrocytes. Following exposure to varying concentrations of exogenous CXCL13 for 12, 24, and 48 h, the data indicated that, in comparison to the control group, both the 200 and 400 ng mL^−1^ treatment groups exhibited a significant reduction in transendothelial electrical resistance (TEER) values (**Figure** **4**A), along with a marked increase in permeability (Pe) coefficients (Figure 4B). Following a 24 h treatment with 200 ng mL^−1^ CXCL13, the viability of bEnd.3 cells were significantly reduced (Figure 4C,D). Furthermore, CXCL13 exposure led to a dose‐dependent down‐regulation or loss of Occludin and ZO‐1 expression in bEnd.3 cells (Figure 4E–G). These findings indicate that CXCL13 compromises tight junction integrity within the BSCB model.

### 2.5 CXCL13 Promoted Autophagy in Endothelial Cells

**Figure 5:**
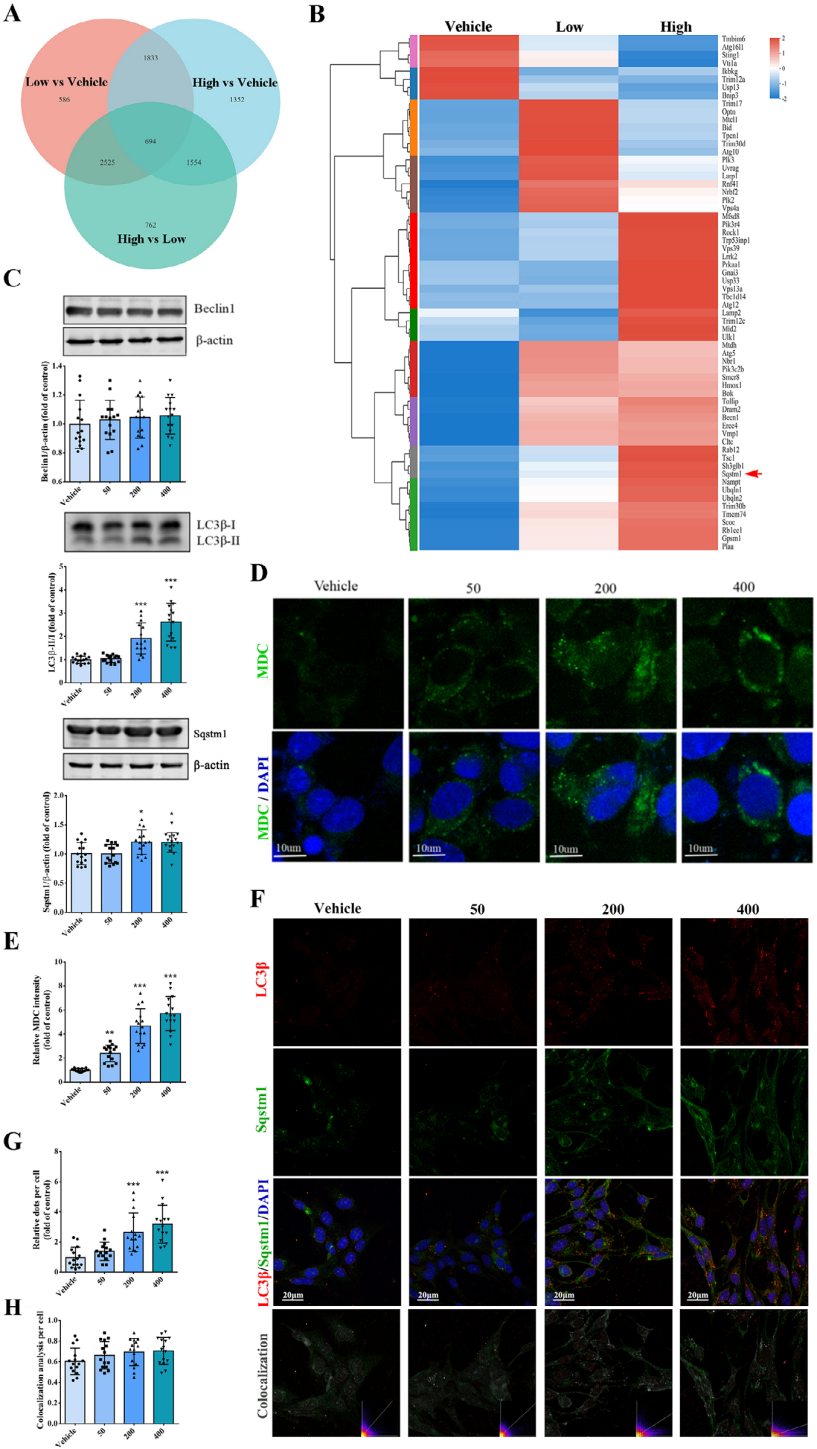
CXCL13 promotes autophagy in bEnd.3 cells. Various concentrations of CXCL13 (0, 50, 400 ng mL^−1^) were introduced into the BSCB culture medium for 24 h. A) Venn diagram shows the number of differentially expressed mRNAs in bEnd.3 cells among ctrl (0 ng mL^−1^), low (50 ng mL^−1^ CXCL13) and high (400 ng mL^−1^ CXCL13) groups. B) Clustered heat map shows the autophagy‐related differentially expressed mRNAs in bEnd.3 cells. Arrow shows Sqstm1. C, Western blot and quantification analyses of Beclin1, LC3β, and Sqstm1 in bEnd.3 cells. D) Autophagy was detected by MDC in bEnd.3 cells. E) Quantification analyses of MDC intensity in D. F) Immunofluorescence staining of LC3β and Sqstm1 in bEnd.3 cells. G) Quantification analyses of puncta in F. H) Colocalization analyses in F. *n*  = 15. Data are presented as the mean ± SD. *p <0.05, **p <0.01, ***p < 0.001 versus Vehicle.

To explore the specific mechanisms by which CXCL13 induces damage in endothelial cells, bEnd.3 cells were exposed to either 50 or 400 ng mL^−1^ of CXCL13 for a duration of 24 h, followed by transcriptome sequencing analysis. The pairwise comparative analysis revealed 694 differentially expressed genes (**Figure** **5**A), among which 56 genes were identified as autophagy‐related and exhibited elevated expression levels in the CXCL13‐treated group (Figure 5B). Notably, the expression of autophagy markers LC3β‐II/I and Sqstm1 was significantly up‐regulated in bEnd.3 cells subjected to the higher concentration of CXCL13 (Figure 5C). MDC labeling demonstrated a marked increase of autophagosomes in response to CXCL13 treatment in a dose‐dependent manner (Figure 5D,E). Furthermore, after treatment with a higher concentration of CXCL13, the cells exhibited significantly increased LC3β^+^ Sqstm1^+^ puncta (Figure 5F–H).

**Figure 6:**
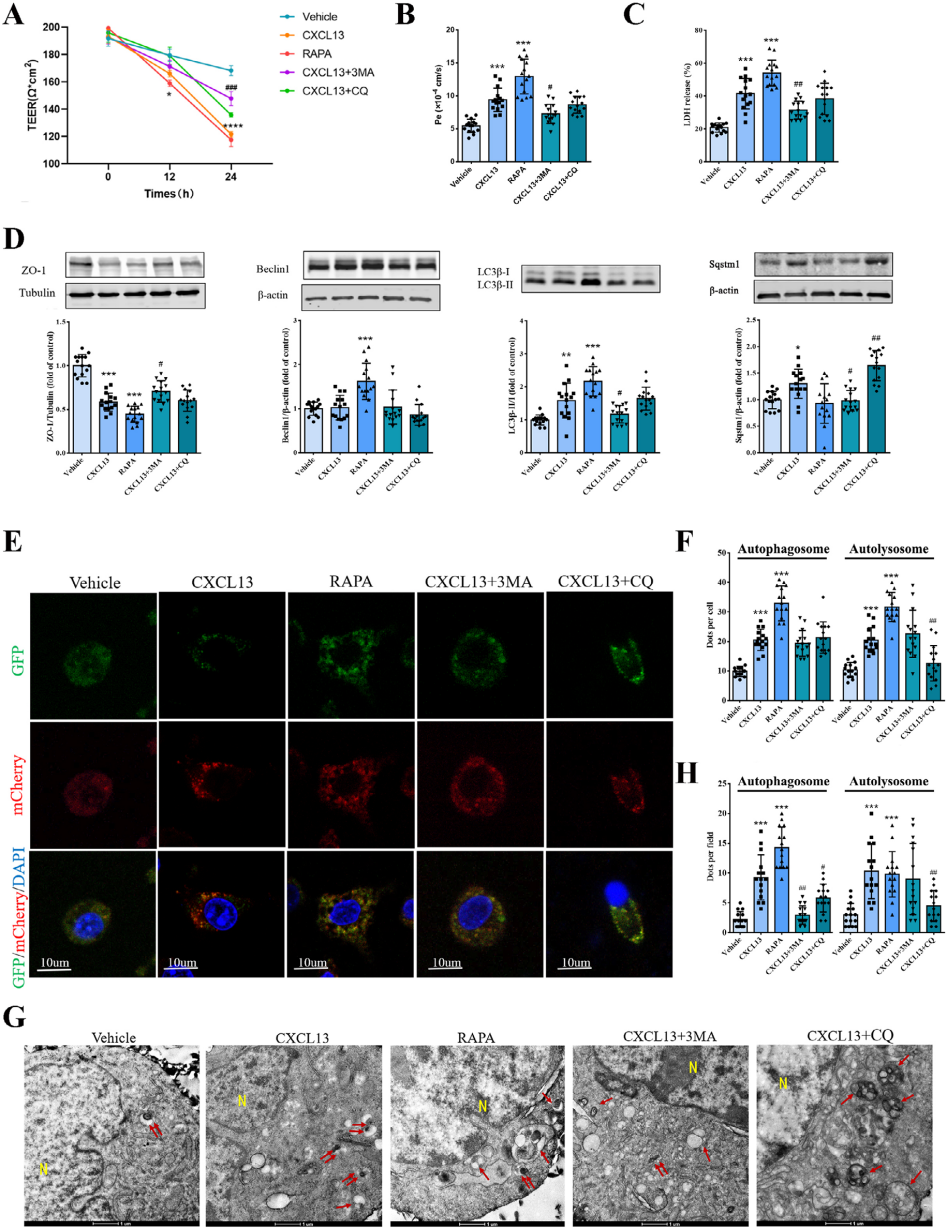
Autophagy inhibitors attenuate the damaging effect of CXCL13 on the BSCB in vitro. 400 ng mL^−1^ CXCL13 was introduced into BSCB culture medium with or without autophagy inhibitor (3MA, CQ) for 24 h. Autophagy agonist Rapamycin (RAPA) was used as a control. A) TEER values are recorded. B) Pe is measured. C) LDH release test for cell damage analysis. D) Western blot and quantification analyses of ZO‐1, Beclin1, LC3β, and Sqstm1 in bEnd.3 cells. E) Images show GFP and mCherry fluorescence. Yellow puncta indicate autophagosomes and red puncta represent autolysosomes. F) Quantification analyses of puncta in E. G) TEM images show the autophagosomes and autolysosomes in bEnd.3 cells. N indicates nuclei. Single arrows represent autophagosomes, double arrows indicate autophagosomes. H) Quantification analyses of autophagosomes and autophagosomes G. *n*  = 15. Data are presented as the mean ± SD. *p < 0.05, **p < 0.01, ***p < 0.001, ****p < 0.0001 versus Vehicle*. ^#^p <*0.05, *^##^p <*0.01 versus CXCL13.

To verify the deleterious impact of CXCL13 in relation to autophagy, autophagy inhibitors, 3MA and CQ, were employed. The findings indicated that, in comparison to the CXCL13 group, the introduction of these inhibitors led to an elevation in TEER values (**Figure** **6**A), a reduction in Pe coefficients (Figure 6B), and a decrease in LDH release (Figure 6C), collectively suggesting diminished cytotoxicity. Furthermore, the inhibitors were observed to down‐regulate the expression of autophagy markers while up‐regulating the expression of ZO‐1 (Figure 6D). Subsequently, autophagic flux was assessed using GFP‐mCherry‐LC3 puncta and TEM. As illustrated in Figure 6E–H, CXCL13 markedly augmented the quantity of autophagosomes and autolysosomes. This increase was substantially diminished upon the addition of autophagy inhibitors in comparison to the CXCL13‐treated group. These findings suggest that CXCL13 facilitates autophagy in endothelial cells.

### 2.6 CXCL13 Upregulated RNF6 to Ubiquitinate Sqstm1

**Figure 7:**
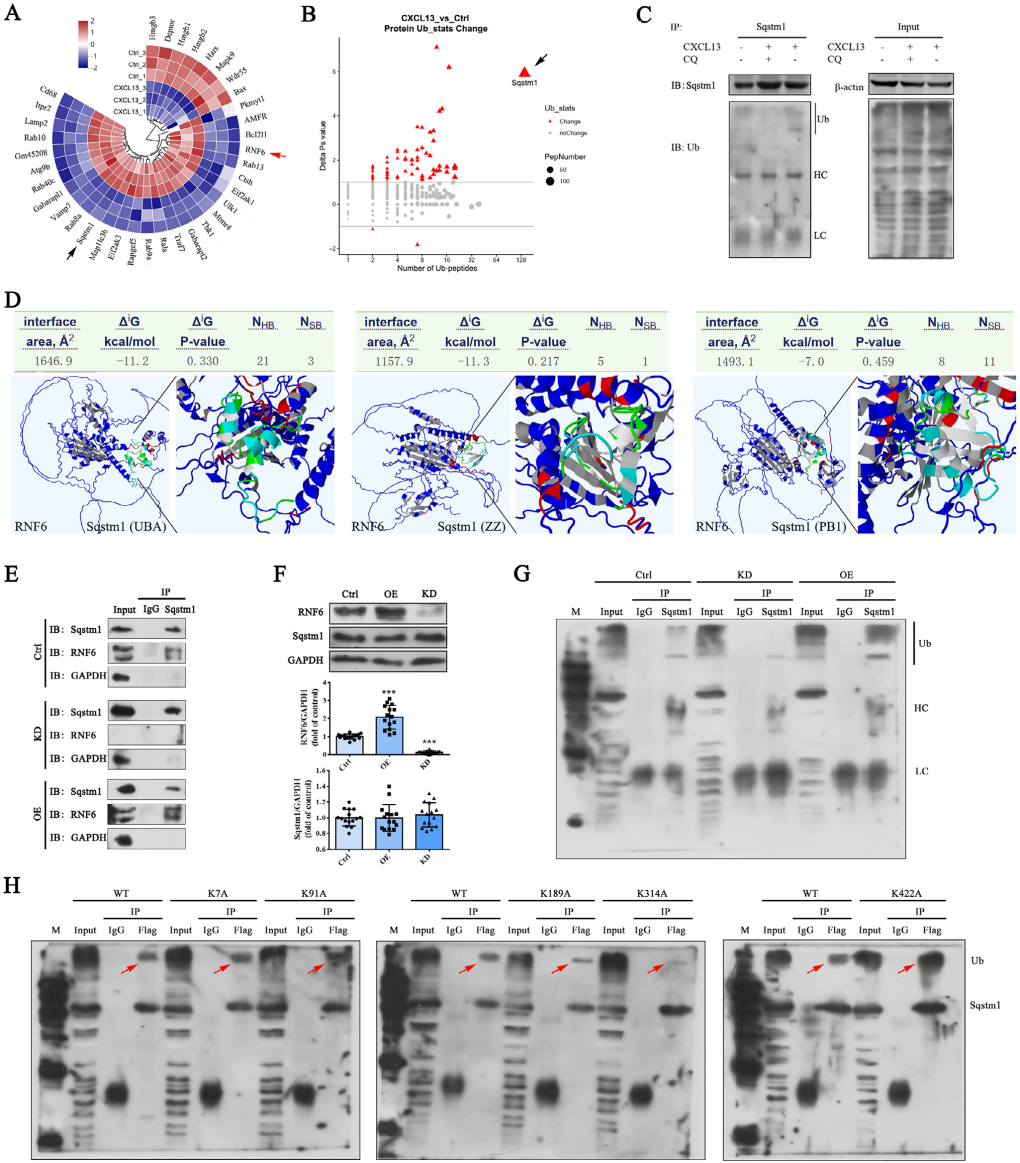
CXCL13 up‐regulates RNF6 for Sqstm1 ubiquitination. 400 ng mL^−1^ CXCL13 was introduced into BSCB culture medium for 24 h. In some trials, autophagy agonist (RAPA) and inhibitor (CQ) were used. A) Quantitative proteomics analysis of differentially expressed autophagy‐related proteins in bEnd.3 cells treated with CXCL13 or without (Ctrl). The red arrow shows RNF6 and the black arrow displays Sqstm1. B) Protein ubiquitination modification mass spectrometry shows differential ubiquitinated peptide segments. Arrow indicates ubiquitinated Sqstm1 peptide segments. C) The ubiquitylation level of Sqstm1 is evaluated by Co‐IP. HC, heavy chain. LC, light chain. D) Molecular docking of RNF6 and different domains of Sqstm1. Interface area in Å^2^ is calculated as the difference in total accessible surface areas of isolated and interfacing structures divided by two. Δ^i^G indicates the solvation‐free energy gain upon formation of the interface, in kcal M^−1^. Δ^i^ *G* P‐value indicates the P‐value of the observed solvation‐free energy gain. *p <*0.5 indicates interfaces with surprising (higher than would‐be‐average for given structures) hydrophobicity, implying that the interface surface can be interaction‐specific. N_HB_ indicates the number of potential hydrogen bonds across the interface. N_SB_ indicates the number of potential salt bridges across the interface. E) Co‐IP assay confirms the interplay between RNF6 and Sqstm1. KD, RNF6 knock down. OE, overexpression of RNF6. F) Western blot and quantification analyses of RNF6 and Sqstm1 in bEnd.3 cells. G, The ubiquitylation level of Sqstm1 is evaluated by Co‐IP in RNF6‐overexpressed or ‐silenced bEnd.3 cells. H) The ubiquitylation level of Sqstm1 is evaluated by Co‐IP in bEnd.3 cells co‐transfected with RNF6 and Sqstm1‐Flag (WT, K7A, K91A, K189A, K314A, and K422A). The red arrow shows ubiquitinated Sqstm1. *n*  = 15. Data are presented as the mean ± SD. ***p < 0.001 versus Ctrl.

To elucidate the mechanism by which CXCL13 regulates autophagy, bEnd.3 cells were exposed to 400 ng mL^−1^ of CXCL13 for a duration of 24 h, followed by protein mass spectrometry analysis. The findings indicated a significant up‐regulation in the expression of Sqstm1 and RNF6, an E3 ubiquitin‐protein ligase, after CXCL13 treatment (**Figure** **7**A). Furthermore, CXCL13 treatment led to a marked increase in the number of ubiquitinated peptides of Sqstm1 (Figure 7B). Subsequently, based on the protein mass spectrometry analysis, a Co‐IP assay was conducted to validate the alterations in ubiquitination levels. In comparison to the control, CXCL13 significantly enhanced the ubiquitination level of Sqstm1, whereas this effect could be inhibited by the application of CQ (Figure 7C).

To assess the potential ubiquitination of Sqstm1 by RNF6, a molecular docking analysis was conducted to predict interactions between RNF6 and various domains of Sqstm1. As illustrated in Figure 7D, RNF6 established hydrogen bonds and salt bridges with amino acid residues across different domains of Sqstm1 (Tables S3–S5, Supporting Information), indicating a stable protein docking model. Subsequently, the interaction between these proteins was corroborated through Co‐IP assays, and the results demonstrated that both RNF6 and Sqstm1 proteins could be immunoprecipitated by the anti‐Sqstm1 antibody, but not by IgG, in bEnd.3 cells. Furthermore, overexpression of RNF6 resulted in an increased amount of immunoprecipitated RNF6 protein (Figure 7E). Notably, although the overexpression or silencing of RNF6 did not influence the expression levels of Sqstm1 (Figure 7F), it did upregulate or downregulate the ubiquitination status of Sqstm1 (Figure 7G).

To further validate the ubiquitination active site of Sqstm1, we conducted an analysis of protein mass spectrometry data, which revealed that the CXCL13‐induced ubiquitinated lysine (K) residues within the Sqstm1 peptide segment were predominantly located at positions 7, 91, 189, 314, and 422. Subsequently, we mutated these lysine residues into arginines and co‐transfected them with RNF6 to assess the ubiquitination status of Sqstm1. The Co‐IP results revealed that the ubiquitination status of the K7, K91, K189, and K422 mutants was comparable to that of the wild‐type Sqstm1. In contrast, the ubiquitination level of the K314 mutant was markedly reduced in the Sqstm1 precipitates (Figure 7H). These findings suggest that CXCL13 enhances the expression of RNF6, which in turn ubiquitinates Sqstm1 specifically at the K314 residue.

## 3 Discussion

MS is an autoimmune degenerative disease characterized by CNS immune cell infiltration and white matter demyelination. BSCB damage is one of the main reasons for inflammatory factors and immune cells entering the CNS to induce neuroinflammation.^[^ ^4^ ^]^ Clinical trials demonstrated that natalizumab, a monoclonal antibody that prevented the infiltration of immune cells into the CNS, reduced the frequency of onset and delayed the progression of MS.^[^ ^15^ ^]^ In order to reveal the mechanism of BSCB damage in MS, this study screened the disease progression‐related cytokine CXCL13 by protein mass spectrometry analysis of peripheral blood serum from clinical MS patients and EAE mice. A growing body of research showed that elevated cerebrospinal fluid and serum levels of CXCL13 were associated with an increased risk of MS progression.^[^ ^11^, ^12^ ^]^ CXCL13 is the most important molecule recruiting B cells to the cerebral spinal fluid for developing lymphoid follicles in the meninges of MS patients. In addition, recent studies have shown that CXCL13 in serum from patients with cerebral metastases could damage the integrity of blood–brain barrier for the metastatic progression of breast cancer into the brain, and LPS‐activated microglial cells might promote invasion and barrier dysfunction in umbilical vein endothelial cells by regulating the CXCL13/CXCR5 axis and p38 signaling.^[^ ^16^ ^]^ Here, using gene knockout mice and in vitro barrier model, it was found that CXCL13 disrupted the integrity of BSCB structure and function, reduced endothelial cell viability, enhanced the expression of E3 ubiquitin ligase RNF6, increased the ubiquitination modification level of Sqstm1, and exacerbated endothelial cell autophagy injury. These results will provide evidence for the study of the pathogenesis of MS and the search for potential prevention and treatment targets.

Many studies have shown that the T help cell 17 (Th17), which originates from the naive T cell, is a key player in the occurrence and development of MS.^[^ ^17^ ^]^ Quinn et al found that in the CNS lesion area of EAE mice induced by Th17 transplantation, only donor‐derived Th17 could be detected at the initial stage. As EAE progresses, a large number of recipient‐derived inflammatory cells, such as follicular helper T cells, Th1, B lymphocytes, etc., infiltrated the CNS, which was called the “second wave of CNS‐infiltration”, eventually leading to demyelination and neurological impairment.^[^ ^18^ ^]^ In addition, studies have shown that Th17 cells were more likely to cross the tight junctions between endothelial cells than other CD4^+^Th cells.^[^ ^19^ ^]^ Thus, it is suggested that Th17 infiltration into the CNS precedes the other immune cells, such as follicular helper cells (Tfh), Th1, B lymphocytes, and so on, during the occurrence and development of EAE.^[^ ^20^ ^]^ It can be inferred that Th17 may be the “initiator” of EAE with a “pioneer” role as: 1) Destroy the BSCB and increase its permeability so as to launch the “second wave of CNS‐infiltration”; 2) Secrete proinflammatory factors to activate astrocytes and microglia in the CNS; 3) Secrete chemokines to recruit immune cells to the lesion areas. Moreover, microarray and ELISA tests revealed that Th17 cells expressed and secreted a large amount of CXCL13, but Th1 cells did not.^[^ ^21^ ^]^ CXCL13 could maintain the activated Th17 lymphocyte response in EAE mice, while CXCL13 neutralizing antibodies effectively inhibited the migration of Th17 and alleviated the inflammatory response.^[^ ^22^ ^]^ Under disease conditions, CXCL13 was mainly secreted by Tfh, dendritic cells, and macrophages in non‐lymphoid tissues. The ectopic CXCL13 expression was involved in the pathogenesis of various immune‐mediated inflammatory diseases as it regulated the migration of B lymphocytes, T lymphocytes, and other immune cells in inflammatory sites as well as influenced the expression of inflammatory factors.^[^ ^23^ ^]^ Based on the above research and our study results, it is concluded that excessive CXCL13 is one of the main reasons for BSCB damage and inflammatory cell infiltration during the development of EAE and MS.

Autophagy, one of the important physiological processes in organisms, meets the metabolic needs of cells and the continuous and dynamic renewal of organelles.^[^ ^24^ ^]^ Autophagy plays an important role in both physiological and pathological processes. Under physiological conditions or metabolic stress conditions such as starvation and hypoxia, autophagy can remove aging and damaged biological macromolecules and organelles in cells, obtain energy sources and materials needed for reconstruction, and maintain normal basic cellular activities. In contrast to the protective role of autophagy in maintaining cellular homeostasis, excessive autophagy will lead to cellular metabolic imbalance and stress response disorder and ultimately induce autophagic cell death.^[^ ^24^, ^25^ ^]^ Studies have shown that autophagy is not only closely related to growth, development, differentiation, and aging but also related to the occurrence and development of tumors, autoimmune diseases, neurological diseases, and cardiovascular and cerebrovascular diseases.^[^ ^24^, ^26^ ^]^ Recent studies found that autophagy played a key role in BSCB structural damage and dysfunction.^[^ ^27^ ^]^ The up‐regulation of autophagic flux in brain microvascular endothelial cells was closely related to the injury and repair of barriers in patients with acute cerebral infarction hemorrhage and a mouse model of middle cerebral artery occlusion/reperfusion.^[^ ^28^ ^]^ Autophagy blocked the depolarization of claudin5 and protected the barriers from hypoxia,^[^ ^29^ ^]^ while autophagy deficiency led to tight junction protein loss and cerebrovascular barrier dysfunction.^[^ ^30^ ^]^ Autophagy also alleviated the glucose and oxygen deprivation reperfusion injury of brain microvascular endothelial cells by reducing the release of reactive oxygen species and inhibiting cell apoptosis.^[^ ^31^, ^32^ ^]^ On the other hand, glucose oxygen deprivation‐induced excessive autophagy in endothelial cells, enhanced autophagy‐lysosome activity, degraded tight junction proteins and resulted in barrier structural damage.^[^ ^33^ ^]^ In the cerebral ischemia‐reperfusion mice model, autophagy blocking exerted a neuroprotective effect through the activation of Sirt1‐FOXO1 signaling pathway.^[^ ^34^ ^]^ Therefore, physiological autophagy can protect cells from a variety of stress injuries, while excessive pathological autophagy leads to endothelial cell dysfunction and even death. Our results show that excessive CXCL13 induces pathological autophagy in endothelial cells, resulting in damage and dysfunction of BSCB.

Sqstm1 is an important selective autophagy adaptor protein, containing 440 amino acids to form several functional regions, such as ubiquitin‐binding domain (UBA), ZZ‐type zinc finger domain, and PB1 domain.^[^ ^35^ ^]^ Dysregulation of autophagy or abnormal Sqstm1 function will lead to blockage of multiple signaling pathways and extreme protein aggregation, resulting in the occurrence of neurodegenerative diseases, diabetes, cancers, etc.^[^ ^36^ ^]^ The regulation of Sqstm1 activity is associated with certain post‐translational modifications, such as phosphorylation, ubiquitination, and so on.^[^ ^37^ ^]^ In recent years, studies have reported that different kinds of E3 ubiquitin ligases mediate ubiquitination of Sqstm1 at specific sites to promote or inhibit its function. RNF26 played a role in EGFR‐driven cell proliferation by ubiquitinating Sqstm1,^[^ ^38^ ^]^ while mutation of Sqstm1 at K420 led to abnormal ubiquitination and thus inhibited autophagy.^[^ ^39^ ^]^ TRIM21 could bind to the PB1 domain to ubiquitinate K7, hindering the oligomerization of Sqstm1 and inhibiting its function.^[^ ^40^ ^]^ CUL5‐ASB6 complex promoted the ubiquitination of Sqstm1 for degradation to inhibit cell proliferation and autophagy.^[^ ^41^ ^]^ In addition, RNF166 and NEDD4 activated autophagy through ubiquitination of Sqstm1.^[^ ^42^ ^]^ KEAP1‐CUL3 ubiquitinated Sqstm1 at the site of K420 within the UBA domain, improved the efficiency of substrate transport to the autophagosome, enhanced selective autophagic flux, and accelerated the degradation of target proteins.^[^ ^43^ ^]^ The ubiquitin‐specific peptidase USP8 acted as a negative regulator of autophagy by deubiquitinating Sqstm1 at K420.^[^ ^44^ ^]^ The deubiquitinating enzyme OTUD7B interacted with IRF3 to remove the polyubiquitin chain at the K7 site of Sqstm1 to enhance the oligomerization for the block of antiviral immune response.^[^ ^45^ ^]^ In this study, we found that RNF6, an E3 ubiquitin ligase, mediated the ubiquitination of Sqstm1 at K314 to promote autophagy.

Protein ubiquitination and deubiquitination, characterized by universality of occurrence, diversity of structure, complexity of regulation, and importance of function, are in a dynamic balance to participate in protein‐specific degradation and regulate cellular signaling pathways.^[^ ^46^ ^]^ At present, it is known that the number of genes encoding ubiquitin‐activating enzyme E1, ubiquitin‐conjugating enzyme E2, ubiquitin ligase E3, and deubiquitinating enzyme is far more than the number of genes responsible for protein phosphorylation‐related kinases and phosphatases in cells, indicating the importance and the complexity of regulation in this post‐translational ubiquitination modification.^[^ ^47^ ^]^ The complexity of ubiquitination regulation is mainly manifested as: 1) macro‐heterogeneity, the differences in ubiquitination of different lysine sites in protein; 2) micro‐heterogeneity, the modification of homogenous or heterologous ubiquitin chains at the same lysine site. In short, future research on protein ubiquitination modification will provide new ideas for the pathogenesis and treatment strategies of many diseases.

This study demonstrates the role and mechanism of CXCL13/RNF6/Sqstm1‐ubiquitination axis in inducing autophagic damage of BSCB endothelial cells during EAE. However, a few possible limitations of our findings could be the lack of a therapeutic schedule for EAE based on reducing CXCL13, as well as unclear crosstalk between CXCL13 and RNF6. Previous evidence suggested that downregulating CXCL13 had a good alleviating effect on MS. For example, CXCL13 levels were reduced in MS at 6 months following mesenchymal stem cell treatment for induction of neuroprotection and neuro‐regeneration.^[^ ^48^ ^]^ Moreover, some main active components of medicinal plants played a remarkable role in reducing the overexpression of CXCL13 and promoting the protein expression of tight junctions to inhibit the cytokine‐cytokine interaction pathways and modulate barrier structure and function.^[^ ^49^ ^]^ In addition, the special focus on the interaction between CXCL13 and its receptor, the G‐protein coupled receptor CXCR5, to build a signaling network that regulates intracellular ubiquitination levels will guide our future research.

## 4 Experimental Section

### Clinical Subjects

Eight patients who were diagnosed with MS for expanded disability status scale measures were recruited from the Fourth People's Hospital of Yancheng and the Affiliated Hospital of Xuzhou Medical University.^[^ ^14^ ^]^ Peripheral blood samples were obtained from MS patients together with age‐ and sex‐matched healthy volunteers, both of whom were informed consenters. This study was approved by the Clinical Research Ethics Committee (NO. YSYLL2021012).

### Mice

C57BL/6 WT mice were obtained from the Experimental Animal Center of Jiangsu Medical College. CXCL13^−/−^ mice were purchased from the Shanghai Model Organisms Center (Shanghai, China). The genotype was identified by polymerase chain reaction with the following primers, TGGTGGTGGCAGCAAAGTTA, TCCTTCCAGAAGCTCTGTGC. The plasma levels of CXCL13 were measured by enzyme‐linked immunosorbent assay (Jianglaibio, China). All experiments were performed in accordance with the Provisions and General Recommendations of the Chinese Experimental Animal Administration Legislation, as well as institutional approval from the Experimental Animal Ethics Committee of Jiangsu Medical College (NO. XMLL2023006).

### BSCB Model and Barrier Properties In Vitro

Mouse brain microvascular endothelial cell line bEnd.3 was purchased from Pricella Biotechnology Co., Ltd (Wuhan, China) and cultured in DMEM supplemented with 10% FBS. Primary astrocytes were extracted from P2 mouse pups. Briefly, the cerebral cortex was digested by trypsin and the cell suspension was cultured in DMEM/F‐12 (1:1) with 10% FBS and L‐glutamine. After 8 days of cultivation, purified astrocytes were isolated by horizontal shaking at 200 r min^−1^ for 18–20 h, following 3 h‐shaking to remove microglia.

For the BSCB model in vitro, bEnd.3 endothelial cells and astrocytes were separately seeded on the upper surface and bottom side of the collagen‐coated transwell insert filters (Corning, pore size: 0.4 µm). TEER values were recorded for measure of junctional tightness as:

(1) $$TEER=\left(Rt-Rb\right)\times A$$

where Rt and Rb represented the total resistance and background resistance, respectively, and A was the area of the transwell. For the fluorophore transmittance trial, 100 µg mL^−1^ FITC‐Dextran was added into the upper chamber of the transwell, followed by shaking in the dark at 100 r min^−1^ for 2 h, then the fluorescence intensity of the culture medium was measured to analyze the Pe value:

(2) $$Pe=\left(dQ/dt\right)/\left(AC_{0}\right)$$

where *dQ/dt* was the transport mass per unit time (g min^−1^), A was the area of the membrane, and C_0_ was the initial concentration of FITC‐Dextran.

In some trials, different doses of CXCL13 (catalog number: 250‐24, PeproTech, USA), Rapamycin (RAPA), Chloroquine (CQ), and 3‐methyladenine (3MA) were added to the culture medium.

### EAE Induction

Eight‐week‐old female mice were immunized subcutaneously with 100 µg MOG_35–55_ (MEVGWYRSPFSRVVHLYRNGK) (catalog number: 51716, GL Biochem, Shanghai, China) in complete Freund's adjuvant (Sigma–Aldrich, St. Louis, MO, USA) containing 4 mg mL^−1^ heat‐killed Mycobacterium tuberculosis H37Ra (catalog number: 231141, BD Bioscience, San Jose, CA, USA). Each mouse was intraperitoneally administered 200 ng pertussis toxin (catalog number: 70323‐44‐3, Sigma–Aldrich, USA) on the day of immunization and 48 h later. All mice were weighed daily to analyze the body‐weight ratio. Clinical assessment of EAE was performed daily as following criteria: 0, no clinical signs; 1, paralyzed tail; 2, paresis (weakness, incomplete paralysis of one or two hindlimbs); 3, paraplegia (complete paralysis of both hindlimbs); 4, paraplegia with forelimb weakness or paralysis; and 5, moribund state or death. For the fluorescein leakage test in vivo, the mouse was administrated with 0.2 mg FITC‐Dextran by intravenous injection, and 40 min later, the mouse was euthanized for sample collection.

### Plasmid Construction and Transfection

RNF6 CDS sequence (CCDS9316.1) and RNF6 shRNA sequences (CCGG CGGGTAGAAACAGGTCTGTTACTCGAGTAACAGACCTGTTTCTACCCGTTTTTG, CCGGGCTCACTTCTTCTTACTGAATCTCGAGATTCAGTAAGAAGAAGTGAGCTTTTTG, CCGGCGAGTTTCACATTCACTGTATCTCGAGATACAGTGAATGTGAAACTCGTTTTTG) were synthesized and cloned into the pIRES‐EGFP and pLKO.1 vectors, respectively. Sqstm1‐Flag (WT, K7A, K91A, K189A, K314A, and K422A) were cloned into the pLV3‐CMV‐CopGFP‐Puro vectors. Plasmids were transfected into bEnd.3 cells using EZ Trans Plus kit (catalog number: AC04L011, Life‐iLab, China).

### Histological and Cellular Staining

After anesthesia, mice were perfused with buffered 4% paraformaldehyde. The paraffin‐embedded spinal cord sections were stained with hematoxylin and eosin (H&E) and Luxol fast blue (LFB).

For histological and cellular immunostaining, paraffin sections, cryosections, or polyformaldehyde‐fixed cells were blocked with goat serum, and then incubated with corresponding primary antibodies (Proteintech, Wuhan, China): CD31 (dilution:1:200, catalog number:11265‐1‐AP), ZO‐1 (dilution:1:500, catalog number:21773‐1‐AP), CXCL13 (dilution:1:200, catalog number:10927‐1‐AP), Iba‐1 (dilution:1:500, catalog number:10904‐1‐AP), LC‐3β (dilution:1:200, catalog number:18725‐1‐AP), Sqstm1 (dilution:1:1000, catalog number:18420‐1‐AP). Enzyme‐labeled secondary antibody or fluorescent secondary antibody was used. Images were acquired using a panoslice scanner (Olympus, Japan) or confocal laser scanning microscope (Leica, Germany) and analyzed by Image J.

### Transmission Electron Microscopy

The cell precipitation cluster was fixed and embedded for the ultrathin section. After staining with uranyl acetate and lead citrate, electron micrographs were captured using an FEI Tecnai G2 T12 transmission electron microscope (Thermo Fisher Scientific, Waltham, MA, USA) and analyzed by TEM Imaging and Analysis software.

### Transcriptomics and Proteomics

Total RNA extracted from bEnd.3 cells and proteins were isolated from bEnd.3 cells and peripheral blood serum of mice and clinical subjects were measured in Majorbio Biopharm Technology Co., Ltd. (Shanghai, China). The data were analyzed on the free online platform of the Majorbio Cloud Platform (www.majorbio.com).

### Flow Cytometry

Type I collagenase‐digested spinal cords from mice were collected to prepare lymphocytes by Percoll gradient centrifugation. For detection of intracellular IL‐17, cells were incubated with Cell Stimulation Cocktail (catalog number: 00‐4970‐03, eBioscience, USA) for 5 h, resuspended in fixation/permeabilization solution (BD Pharmingen, USA) and stained with IL‐17 and CD4 antibodies (dilution:1:50, catalog number: 130‐123‐799, 130‐116‐509, Miltenyi Biotec, Germany). Labeled cells were measured on MACSQuantTM Flow Cytometers (Miltenyi Biotec, Germany) and analyzed with FlowJo software.

### CCK‐8 Assay

Cells were planted on 96‐well plates, and then CCK−8 solution was added into each well of different groups for 4 h incubation. Finally, the optical density was measured at 450 nm.

### LDH Release Assay

LDH release assay was performed using a commercial kit (catalog number: C0018 M, Beyotime, China). Briefly, the cells were washed with PBS and the culture medium was replaced before the experiment. The background blank control group and maximum enzyme activity control group were established. A mixed solution consisting of 120 µL of supernatant from each group and 60 µL of work solution was prepared for the measurement of absorbance at 490 nm. The LDH release ratio was:

(3) $$\left(OD_{sample}-OD_{blank}\right)/\left(OD_{max}-OD_{blank}\right)$$

### Western Blotting

The prepared protein samples from tissues or cells were separated by electrophoresis and transferred to nitrocellulose membranes. After blocking, the membranes were incubated overnight at 4 °C with primary antibodies (Proteintech, China): Occludin (dilution:1:20 000, catalog number:27260‐1‐AP), ZO‐1 (dilution:1:10 000, catalog number:21773‐1‐AP), LC‐3β (dilution:1:1000, catalog number:18725‐1‐AP), Sqstm1 (dilution:1:10 000, catalog number:18420‐1‐AP), Beclin1 (dilution:1:3000, catalog number:11306‐1‐AP), RNF6 (dilution:1:1000, catalog number:20437‐1‐AP), Ubiquitin (dilution:1:3000, catalog number:10201‐2‐AP), GAPDH (dilution:1:100 000, catalog number:60004‐1‐Ig), Tubulin (dilution:1:100 000, catalog number:66240‐1‐Ig), β‐actin (dilution:1:50 000, catalog number:66009‐1‐Ig), and then incubated in IRDye‐conjugated secondary antibodies (LI‐COR, USA). Membranes were scanned using an Odyssey Infrared Imaging System Scanner (LI‐COR, USA), and images were analyzed using ImageJ software.

### Autophagy Detection Assay

According to the kit manual (catalog number: C3018S, Beyotime, China), the culture medium was replaced with Monodansylcadavrine (MDC) staining solution for 30 min‐incubation at 37 °C. After washing, the cells were detected using a confocal laser scanning microscope.

### Autophagic Flux Measurement

Cells were infected with lentivirus carrying mCherry‐GFP‐LC3β (catalog number: D2816, Beyotime, China) at MOI of 10. After virus‐containing medium discarding and positive screening, the cells were subjected to different treatments. Finally, the nucleus was stained with DAPI and cells were observed under a confocal laser scanning microscope. Data analysis was performed using Image J.

### Molecular Docking Prediction

Rigid protein–protein docking (ZDOCK) was performed between RNF6 and Sqstm1 to study the relationships in the zdock server website (https://zdock.wenglab.org/). The PDB formats of the Sqstm1 structural domain, UBA (2KNV), PB1 (6JM4), and ZZ (5YP7), as well as computed structure model RNF6 (AF‐Q9Y252‐F1), were downloaded from the Protein Data Bank PDB database (http://www.rcsb.org/). The PDBePISA (https://www.ebi.ac.uk/pdbe/pisa/) was operated to identify the docking sites and calculate the ZDOCK scores.

### Co‐Immunoprecipitation

Cells were harvested, lysed, sonicated, and then part of the supernatant from the cell lysis solution was used as the input. The remaining portion was incubated with A/G Agarose (Beyotime, China) and 2 µg corresponding antibodies (Sqstm1, catalog number:18420‐1‐AP; Flag, catalog number:66008‐4‐Ig, Proteintech, China): at 4 °C overnight. Subsequently, after washing, the centrifugal sediment was boiled at 95 °C for 15 min. Finally, the supernatant was collected for immunoblotting and subsequent analysis. IgG was used as a control for immunoprecipitation.

### Statistical Analysis

Each experiment was repeated three times at least. Statistical analysis was carried out with GraphPad Prism. All results were summarized and presented as the mean ± standard deviation (SD). Independent sample t‐tests were used to evaluate the differences between groups. One‐way analysis of variance (ANOVA) followed by Bonferroni's post hoc test was used for multiple comparisons. EAE clinical scores were evaluated by two‐way repeated‐measures ANOVA. A *P* value of 0.05 or less was considered statistically significant.

### Ethics Approval

All experiments were performed in accordance with the Provisions and General Recommendations of the Chinese Experimental Animal Administration Legislation, as well as institutional approval from the Clinical Research Ethics Committee of Fourth People's Hospital of Yancheng, Affiliated Hospital of Xuzhou Medical University, and Experimental Animal Ethics Committee of Jiangsu Medical College.

## Conflict of Interest

The authors declare no conflict of interest.

## Author Contributions

J.H. and R.H. contributed equally to this work. The design of the project was contributed by J.H., R.H., A.Z., and X.Q.; Experimental operation and data collection were contributed by J.H., R.H., C.C., W.F., W.Z., G.W., C.Z., J.T., and Y.Y.; Data analysis and interpretation were contributed by J.H., G.W., C.Z., A.Z., and X.Q.; Writing and editing were contributed by J.H. and X.Q. This manuscript was approved by all authors for publication. This work described was original research that had not been published previously and was not under consideration for publication elsewhere, in whole or in part.

## Supporting information



Supporting Information

Supporting Information

Supporting Information

## Data Availability

The data that support the findings of this study are available from the corresponding author upon reasonable request.
