# Supplementary material for: CXCL13 Damages Blood Spinal Cord Barrier by Promoting RNF6/Sqstm1‐Ubiquitination Induced Autophagy in Experimental Allergic Encephalomyelitis
Source: Adv Sci (Weinh). 2025 Apr 15;12(21):2414550. doi: 10.1002/advs.202414550 (PMC12140337; doi:10.1002/advs.202414550)
Supplement: Supplementary file 1 — Supporting Information [file ADVS-12-2414550-s003.docx]

Table 3 List of Hydrogen Bonds and Salt Bridges connecting RNF6 and UBA domain of Sqstm1.

| Num. | **Hydrogen bonds** | | | **Salt bridges** | | |
| --- | --- | --- | --- | --- | --- | --- |
|  | RNF6 | Dist. [Å] | Sqstm1 (UBA) | RNF6 | Dist. [Å] | Sqstm1 (UBA) |
| 1 | ARG 160[NH1] | 3.03 | GLN 16[O] | LYS 36[NZ] | 2.59 | GLU 25[OE2] |
| 2 | GLY 161[N] | 2.70 | GLN 16[OE1] | ARG 508[NE] | 3.74 | HIS 52[O] |
| 3 | ASN 509[ND2] | 3.52 | MET 20[O] | ARG 08[NH1] | 3.36 | HIS 52[O] |
| 4 | GLN 507[N] | 2.71 | GLY 21[O] |  |  |  |
| 5 | THR 35[OG1] | 2.63 | GLU 25[OE2] |  |  |  |
| 6 | LYS 36[NZ] | 2.59 | GLU 25[OE2] |  |  |  |
| 7 | ARG 31[NE] | 2.79 | GLY 26[O] |  |  |  |
| 8 | ARG 31[NH2] | 2.74 | GLY 26[O] |  |  |  |
| 9 | HIS 165[N] | 3.50 | TYR 38[O] |  |  |  |
| 10 | TYR 49[OH] | 3.04 | ASP 45[O] |  |  |  |
| 11 | ASP 19[OD2] | 3.13 | SER 2[N] |  |  |  |
| 12 | ASP 19[OD2] | 2.25 | SER 2[OG] |  |  |  |
| 13 | GLU 163[O] | 3.83 | SER 13[OG] |  |  |  |
| 14 | HIS 165[O] | 3.02 | ASP 39[N] |  |  |  |
| 15 | HIS 165[O] | 3.41 | TYR 38[N] |  |  |  |
| 16 | LEU 506[O] | 2.76 | PHE 22[N] |  |  |  |
| 17 | GLN 507[OE1] | 3.44 | SER 23[OG] |  |  |  |
| 18 | GLU 25[OE2] | 2.57 | THR 35[OG1] |  |  |  |
| 19 | GLY 26[O] | 3.03 | ARG 31[NH2] |  |  |  |
| 20 | ASP 45[O] | 2.76 | TYR 49[OH] |  |  |  |
| 21 | TYR 49[OH] | 2.79 | GLN 48[NE2] |  |  |  |

**Dist. [Å]** indicates the distance between bridging atoms in Å.

Table 4 List of Hydrogen Bonds and Salt Bridges connecting RNF6 and ZZ domain of Sqstm1.

| Num. | **Hydrogen bonds** | | | **Salt bridges** | | |
| --- | --- | --- | --- | --- | --- | --- |
|  | RNF6 | Dist. [Å] | Sqstm1 (ZZ) | RNF6 | Dist. [Å] | Sqstm1 (ZZ) |
| 1 | ARG 27[NE] | 3.81 | GLY 130[O] | GLU 26[OE2] | 2.94 | LYS 157[NZ] |
| 2 | GLN 30[NE2] | 2.33 | CYS 131[O] |  |  |  |
| 3 | ARG 27[NH2] | 3.50 | TYR 148[OH] |  |  |  |
| 4 | GLU 26[OE2] | 2.94 | LYS 157[NZ] |  |  |  |
| 5 | CYS 131[O] | 3.81 | LYS 157[NZ] |  |  |  |

**Dist. [Å]** indicates the distance between bridging atoms in Å.

Table 5 List of Hydrogen Bonds and Salt Bridges connecting RNF6 and PB1 domain of Sqstm1.

| Num. | **Hydrogen bonds** | | | **Salt bridges** | | |
| --- | --- | --- | --- | --- | --- | --- |
|  | RNF6 | Dist. [Å] | Sqstm1 (PB1) | RNF6 | Dist. [Å] | Sqstm1 (PB1) |
| 1 | LYS 7[NZ] | 2.92 | ASP 69[OD2] | LYS 7[NZ] | 2.92 | ASP 69[OD2] |
| 2 | ARG 50[NH1] | 2.78 | GLU 81[OE1] | ARG 22[NH1] | 3.94 | ASP 69[OD2] |
| 3 | ARG 21[NH1] | 3.53 | GLU 82[OE1] | ARG 50[NE] | 3.63 | GLU 81[OE1] |
| 4  5 | ARG 21[NH1]  ARG 22[NH2] | 3.35  2.47 | GLU 82[OE2]  TYR 89[OH] | ARG 50[NH1]  ARG 50[NH2] | 2.78  3.61 | GLU 81[OE1]  GLU 81[OE1] |
| 6 | ASN 59[ND2] | 3.73 | LYS 91[O] | ARG 50[NE] | 3.98 | GLU 81[OE2] |
| 7 | VAL 432[O] | 2.89 | ILE 20[N] | ARG 50[NH1] | 3.95 | GLU 81[OE2] |
| 8 | ILE 20[O] | 2.80 | ALA 76[N] | ARG 50[NH2] | 3.35 | GLU 81[OE2] |
| 9 |  |  |  | ARG 21[NH1] | 3.53 | GLU 82[OE1] |
| 10 |  |  |  | ARG 21[NE] | 3.92 | GLU 82[OE2] |
| 11 |  |  |  | ARG 21[NH1] | 3.35 | GLU 82[OE2] |

**Dist. [Å]** indicates the distance between bridging atoms in Å.
